# Determination of whole genome sequence of human cytomegalovirus circulating in Japan and discovery of geographic genome structure in *UL148* gene

**DOI:** 10.1016/j.virusres.2025.199540

**Published:** 2025-02-04

**Authors:** Yuji Wada, Ken Ishioka, Tatsuo Suzutani

**Affiliations:** Department of Microbiology, School of Medicine, Fukushima Medical University, Japan

**Keywords:** Human cytomegalovirus, Phylogenetic analysis

## Abstract

Human cytomegalovirus (HCMV), a member of the Herpesviridae family, is prevalent worldwide. HCMV is generally asymptomatic but causes severe disease in immunocompromised patients or infants who are congenitally infected. Recent advances in sequencing technology have led to the rapid expansion of the HCMV genetic database, providing a comprehensive resource for studying viral genetics. Although genetic investigations have been vigorously performed in European countries, information on the whole-genome sequence of HCMV in the East Asian region remains limited. In this study, we determined whole-genome sequences of two clinical isolates of HCMV circulating in Japan. Partial genome sequences of 26 genes in UL/b' region were also identified using additional seven clinical isolates. Phylogenetic analysis of the *UL148* gene revealed a characteristic genetic clade predominantly constructed from HCMV isolates from Japan and China, suggesting a geographic gene structure in the East Asian region. We consider that this research will contribute to expanding the genetic database of HCMV and unveiling novel genetic characteristics of HCMV in Asia.

## Highlights

• The whole and partial genome sequences of clinical HCMV isolates in Japan were determined.
• We identified a novel form of the *UL148* gene in clinical isolates from Japan and China.
• Phylogenetic analysis targeting the UL/b' region was conducted.
• This research expanded the genetic database of HCMV and unveiled novel geographic gene structures in Asia.

Human cytomegalovirus (HCMV) is a member of the herpesvirus family and possesses approximately 235 kbp of double-stranded DNA as viral genome [1]. HCMV is commonly transmitted worldwide, and their seroprevalences are estimated to range from 66 to 90 % depending on the region or country [1,2]. Most patients show asymptomatic or self-limiting mild symptoms by HCMV infection; however, the virus causes severe disease in immunocompromised patients, such as transplant recipients [1,3]. In cases HCMV infects to or reactivates in pregnant women, fetuses are transplacentally infected known as congenital cytomegalovirus infection (cCMV), and this disease causes a variety of symptoms represented by sensorineural hearing loss at birth or in childhood [4]. Vaccine development has advanced to prevent injury from CMV infection, but no licensed vaccine is available to date [5].

Recently, long-read next-generation sequencers have been developed and popularized in laboratory and clinical settings, and whole-genome sequences of HCMV have been vigorously analyzed [[6], [7], [8]]. The enrichment of HCMV sequence information unveiled geographic gene structures in its genome, which might be beneficial for designing global vaccines [9]. However, most of the complete genome sequences available in the database have been reported in European countries. To investigate the geographical characteristics of the HCMV genome further, it is essential to expand the global genetic database.

Here, we analyzed the whole-genome sequences of clinical isolates of HCMV derived from infants with symptomatic cCMV using nanopore sequencing. Briefly, sequencing libraries were prepared using the SQK-LSK114 ligation sequencing kit (Oxford Nanopore Technology (ONT), Oxford, UK) following the manufacturer's protocol. The libraries were loaded onto FLO-MIN114 and sequenced using MinION Mk1C (ONT). The obtained data were base-called using the Guppy software (ONT). The obtained reads were tentatively mapped onto complete HCMV genome sequences available in GenBank using Minimap2 (version 2.17). The mapped reads were output using SAMtools (version 1.9) and visualized using GENETYX-NGS/MAC (version 5.0.5, GENETYX, Tokyo, Japan). In preliminary analysis of nanopore sequencing, it was observed that several genomic regions of the analyzed-HCMV isolates did not align with the complete HCMV genome sequences deposited in GenBank. To understand whole genome structures of the analyzed-HCMV isolates, tentative consensus sequences were determined by manually connecting the unmapped HCMV genomic regions. The samples were then re-analyzed by nanopore sequencing employing an adaptive sampling method using the tentative consensus sequences as a reference. The adaptive sampling method could efficiently analyze mapped reads with no unfavorable procedures or effects, and enough amounts of data have been obtained to determine whole genome sequences of HCMV [10].

Finally, the complete genome sequences of two HCMV isolates, 22,383 and 20,026 (accession numbers: LC846338 and LC846339), were determined. The obtained data comprised 29,462 mapped reads ranging up to 22,893 nt with an average coverage depth of 70.85 reads/nt in isolate 22,383, and 27,299 mapped reads ranging up to 37,232 nt with an average coverage depth of 44.15 reads/nt in isolate 20,026. Sanger sequencing was performed to confirm the genomic regions where frameshift mutations were suggested by nanopore sequencing and to correct them as needed.

**Fig. 1:**
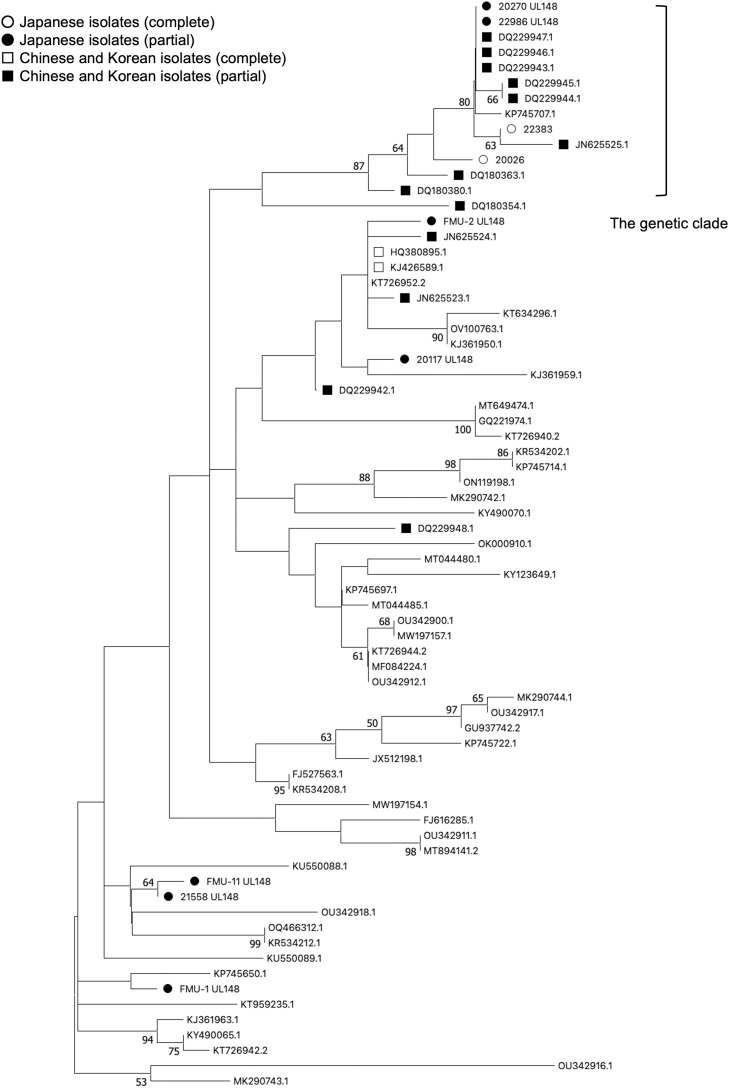
**Phylogenetic analysis of HCMV *UL148* gene.** A phylogenetic tree was constructed using nucleotide sequences of the HCMV *UL148* gene by Maximum Likelihood phylogenetic analysis in the MEGA11 program. A total of 71 HCMV *UL148* sequences (951 bp in length) were used for analysis. The percentage of trees in which the associated taxa clustered together was calculated by 1000 replicates, and the frequencies of >50 % were shown at the branches. The two sequences derived from whole genome sequences of HCMV determined in this study are depicted by white circle. The other seven sequences determined in this study are depicted by black circle. The deposited sequences in GenBank, which were isolated in China and Korea, were depicted by white (complete sequences) and black (partial sequences) squares, respectively.

The sequences of the *UL148* gene in 22,383 and 20,026 were similar to HCMV isolates from China. To investigate the genetic character of the *UL148* gene further, we additionally exploited seven clinical isolates of HCMV, which had also been isolated from infants with symptomatic cCMV in Japan, and the nucleotide sequences of the *UL148* gene were determined (accession numbers are listed in the Supplemental Table). Phylogenetic analysis was performed using MEGA11 software (version 11.0.13) using Maximum Likelihood method and Tamura-Nei model [11]. The phylogenetic tree of the *UL148* gene showed that four of nine analyzed-HCMV isolates (44.4 %) formed a characteristic genetic clade predominantly constructed from clinical isolates in Japan and China (Fig. 1). As an exception, the BE/13/2012 strain (KP745707) isolated in Belgium also belongs to this clade. Charles *et al*. reported that some HCMV strains isolated in Europe were derived from an Asian population; therefore, the BE/13/2012 strain might originate from the Asian region [9]. Alternatively, it would be also possible that Asian type of the *UL148* gene was introduced into origin of the BE/13/2012 strain in Europe through superinfection and recombination event [7,12]. Considering the genetic features of HCMV, it would be understandable some European HCMV strains possessed geographically characteristic gene structures in Asian region. Nucleotide BLAST analysis confirmed that no other exceptional strains belonged to this clade. Based on these observations, the characteristic gene structures in the East Asian region are considered to be conserved in the *UL148* gene.

**Fig. 2:**
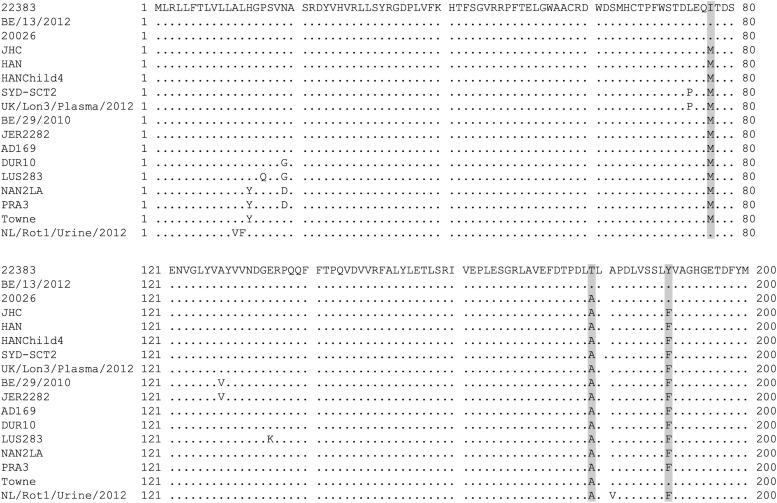
**Amino acid sequence alignment of HCMV UL148.** Multiple alignment analysis of HCMV UL148 are shown. The amino acid sequences used in this analysis were randomly corrected by regions or countries. As exceptions, Towne and NL/Rot1/Urine/2012 strains were manually employed in this analysis, because amino acid BLAST analysis showed these strains possessed M77I and F189Y mutations. Dots indicate identical amino acid. Non-synonymous mutations specific in the genetic clade of the *UL148* gene are highlighted in gray.

Among the HCMVs constructing the genetic clade of the *UL148* gene, 13–15 nucleotide substitutions, including three non-synonymous mutations (M77I, A179T, and F189Y), were observed compared to the HAN (KJ426589) and JHC (HQ380895) strains, whose complete genome sequences were available in GenBank as East Asian HCMV strains (Fig. 2). The M77I and F189Y mutations were common in all strains constructing the genetic clade of the *UL148* gene, but they were also observed in several clade-unrelated strains. Although several HCMV strains possessed the M77I and F189 mutations in UL148, amino acid BLAST analysis confirmed that these mutations were quite uncommon comparing to available HCMV sequences in GenBank. In contrast, the A179T mutation was exclusively observed in the genetic clade of the *UL148* gene, even though it was not observed in 20,026. The *UL148* gene encodes an endoplasmic reticulum-resident glycoprotein, named as UL148, which is involved in the evasion of host immunity and alteration of viral cell tropism through the regulation of envelope glycoprotein expression [13,14]. Based on the role of the UL148 protein, M77I, A179T, and F189Y mutations may have emerged as a result of ethnicity-dependent immunological pressure. To assess whether these amino acid substitutions were positively selected, the dN/dS at the codon level was estimated using the FEL method in the HyPhy package [15,16]. No significant differences were observed in these three codons, suggesting no evidence of positive selection, at least in the statistical analysis (M77I: *p* = 0.846, A179T: *p* = 0.667, and F189Y: *p* = 0.379). Further investigations are needed to understand the role of these mutations, including the biological activity of UL148.

**Fig. 3:**
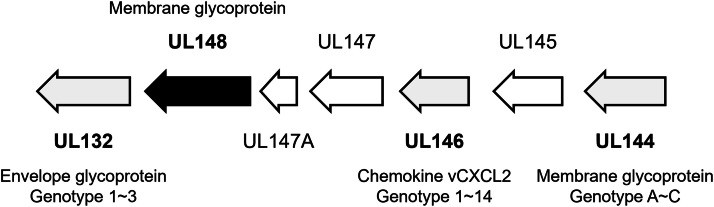
**A diagram of protein-coding ORF in UL/b’ region of HCMV.** The genetic organization between the UL132 gene to the UL144 gene of HCMV are shown. Protein coding region and direction are indicated by arrows. The *UL148* gene is highlighted in black. The genes analyzed their genotype (the *UL132, UL146*, and *UL144* genes) are highlighted in grey.

Finally, the analysis target was expanded to the UL/b' region of HCMV, where the *UL148* gene was located. Besides the *UL148* gene, nucleotide sequences of 25 genes in the UL/b' region were determined using the 7 clinical isolates of HCMV in Japan, and phylogenetic analysis was performed for all of the analyzed-genes (Supplemental Table and Figure). Genetic clades predominantly constructed from HCMV isolates in Japan and China were also observed in the *UL132* and *UL147* genes, which were located surrounding the *UL148* gene. However, these genetic clades were constructed from fewer HCMV isolates in Japan than the *UL148* gene (two of nine analyzed-HCMV isolates) and additional non-Asian HCMV strains. Genetic variabilities were also analyzed by targeting the *UL132, UL146* and *UL144* genes, which have been previously investigated for the genotyping of HCMV (Fig. 3) [[17], [18], [19]]. Various genotype sets were observed in the analyzed-isolates; however, no consistent genotypes were observed among respective pairs of the *UL132, UL146*, and *UL144* genes in 22,383, 22,986, 20,270, and 20,026 isolates (Table 1). Therefore, the genotypes of the *UL132, UL146* and *UL144* genes would not be associated with the M77I, A179T, and F189Y mutations in the *UL148* gene. These observations suggest that the characteristic gene structures in the East Asian region were partially common in *UL148* and the surrounding genes but were mainly conserved in the *UL148* gene. There was low nucleotide diversity and no evidence of recombination in the *UL148* gene [7]. Considering its independence and stability, it is possible that the synonymous and non-synonymous mutations accumulated in the *UL148* gene through local virus circulation and reflected the geographic viral evolution. On the other hand, the phylogenetic analysis of the *UL148* gene showed that five of nine analyzed-HCMV isolates were not classified into the genetic clade and dispersed through the phylogenetic tree with non-Asian HCMVs (Fig. 1). This phenomenon suggested that two types of HCMVs having distinct origins have been circulating in Japan, which were locally evolved one and globally spread one.

**Table 1:** Genotyping analysis of the *UL132, UL146*, and *UL144* genes.

			Genotype (group ID)		
Sample ID	Sequence availability	AA mutations in UL148	*UL132*	*UL146*	*UL144*
22,383	complete	M77I A179T F189Y	2	6	B
22,986	partial		3	12	B
20,270	partial		2	12	C
20,026	complete	M77I F189Y	1	11	B
FMU-1	partial	Not Detected	1	7	A
FMU-2	partial		3	1	C
FMU-11	partial		1	8	C
20,117	partial		3	9	A
21,558	partial		1	1	C

In this study, we determined the whole and partial genome sequences of clinical isolates of HCMV circulating in Japan. We also identified a novel form of the *UL148* gene in clinical isolates from Japan and China. This report will contribute to overcoming the obstacles to the genetic investigation of HCMV, which is limited to whole-genome sequence information of HCMV in the East Asian region.

## CRediT authorship contribution statement

**Yuji Wada:** Writing – review & editing, Writing – original draft, Visualization, Validation, Methodology, Investigation, Funding acquisition, Formal analysis, Data curation, Conceptualization. **Ken Ishioka:** Writing – review & editing, Resources. **Tatsuo Suzutani:** Writing – review & editing, Supervision, Resources, Project administration.

## Declaration of competing interest

The authors declare that they have no known competing financial interests or personal relationships that could have appeared to influence the work reported in this paper.

## Data Availability

Data will be made available on request.

## Appendix Supplementary materials

Image, application 1

Image, application 2

Image, application 3
