# Supplementary material for: Determination of whole genome sequence of human cytomegalovirus circulating in Japan and discovery of geographic genome structure in UL148 gene
Source: Virus Res. 2025 Feb 4;353:199540. doi: 10.1016/j.virusres.2025.199540 (PMC11846927; doi:10.1016/j.virusres.2025.199540)
Supplement: Supplementary file 1 [file mmc1.pptx]

## Slide 1
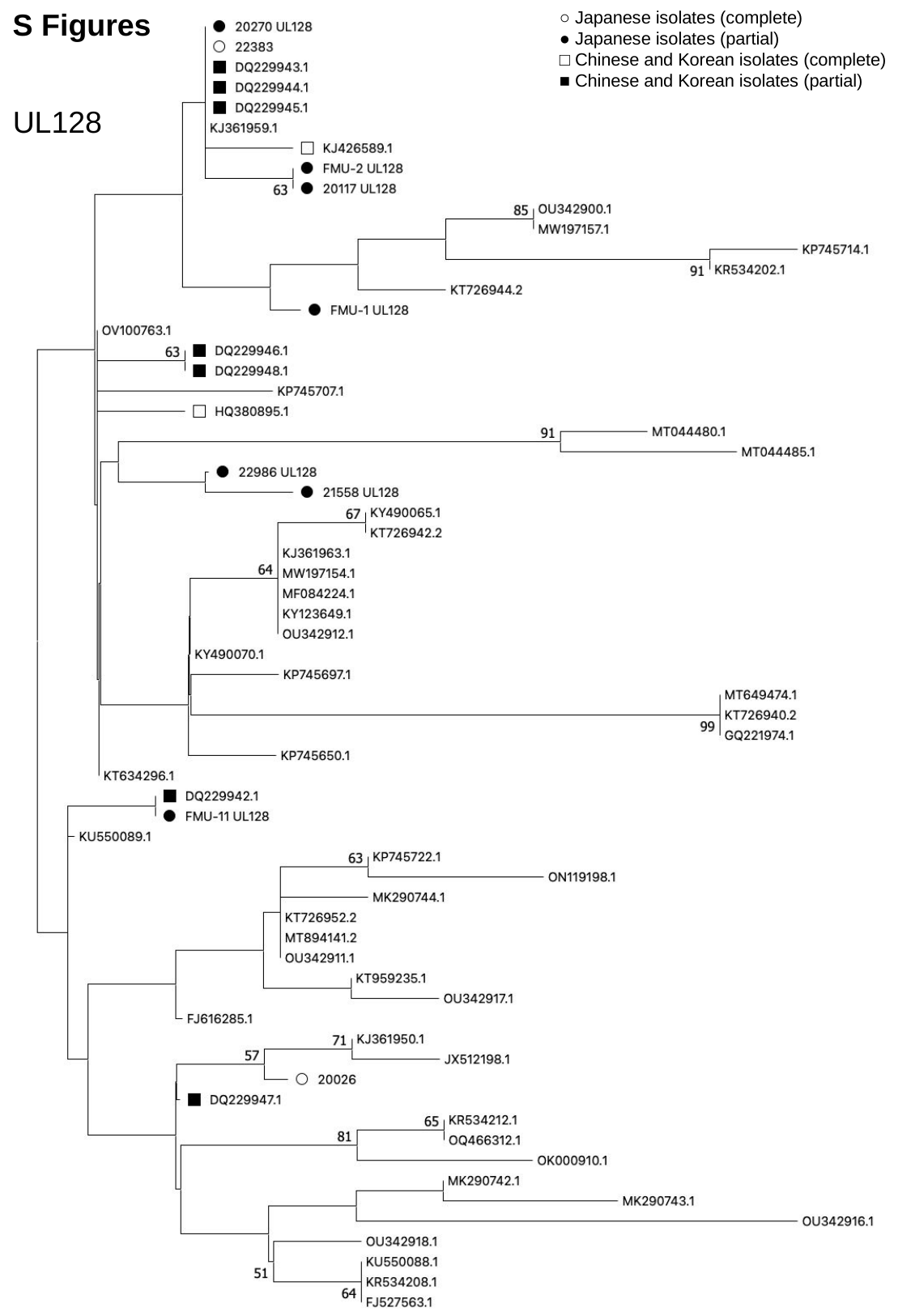

S Figures
○ Japanese isolates (complete)
● Japanese isolates (partial)
□ Chinese and Korean isolates (complete)
■ Chinese and Korean isolates (partial)
UL128

## Slide 2
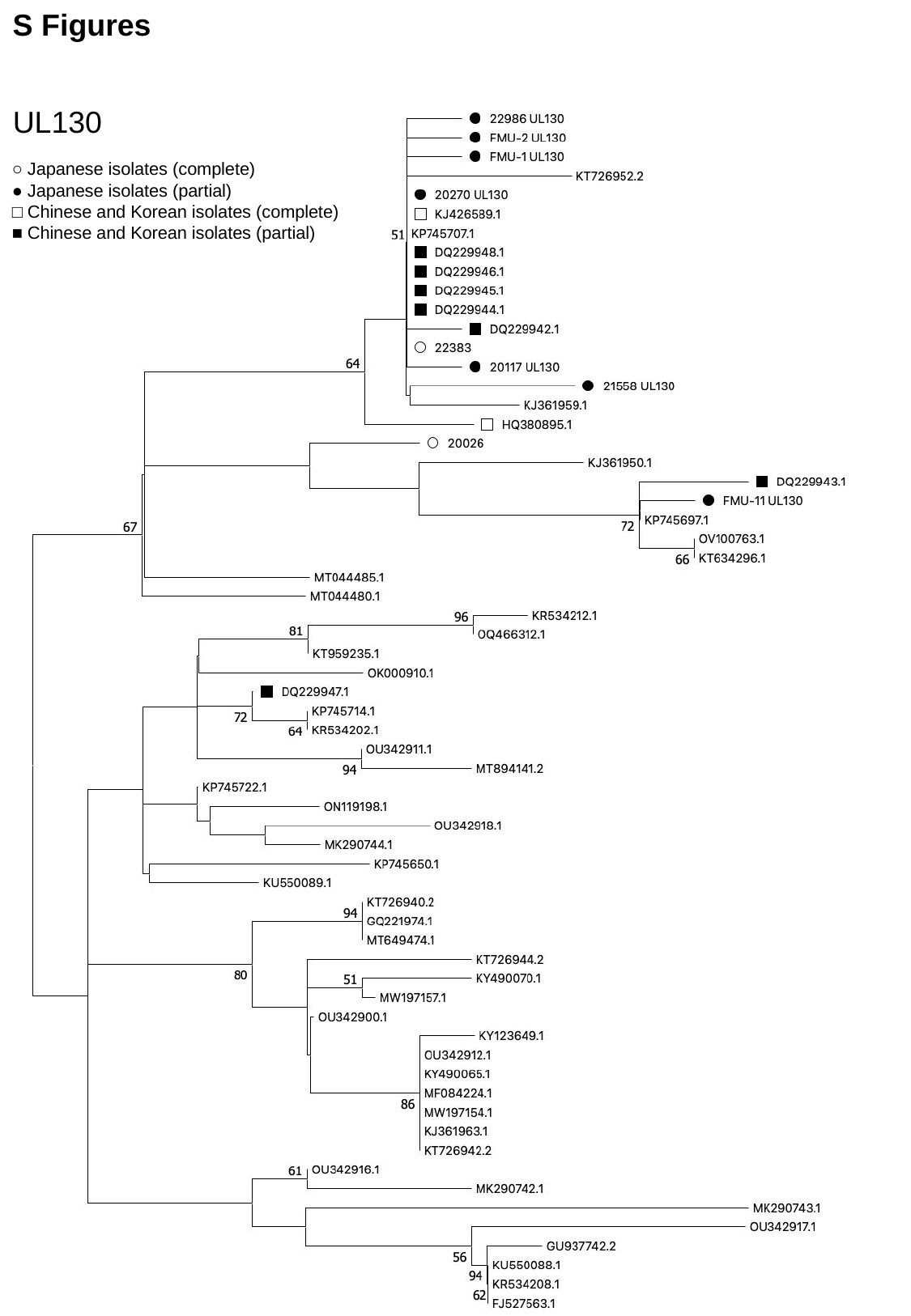

S Figures
UL130
○ Japanese isolates (complete)
● Japanese isolates (partial)
□ Chinese and Korean isolates (complete)
■ Chinese and Korean isolates (partial)

## Slide 3
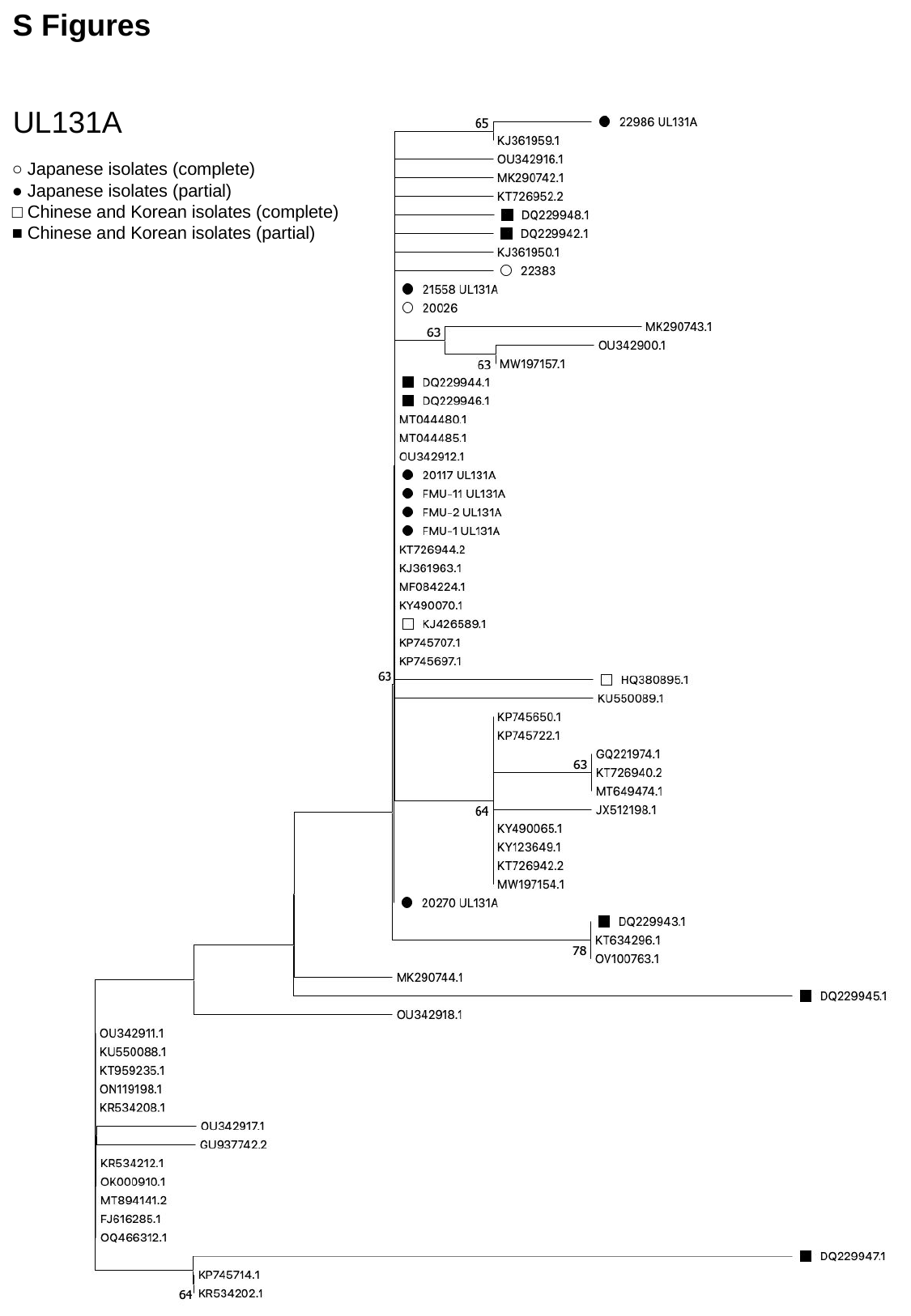

S Figures
UL131A
○ Japanese isolates (complete)
● Japanese isolates (partial)
□ Chinese and Korean isolates (complete)
■ Chinese and Korean isolates (partial)

## Slide 4
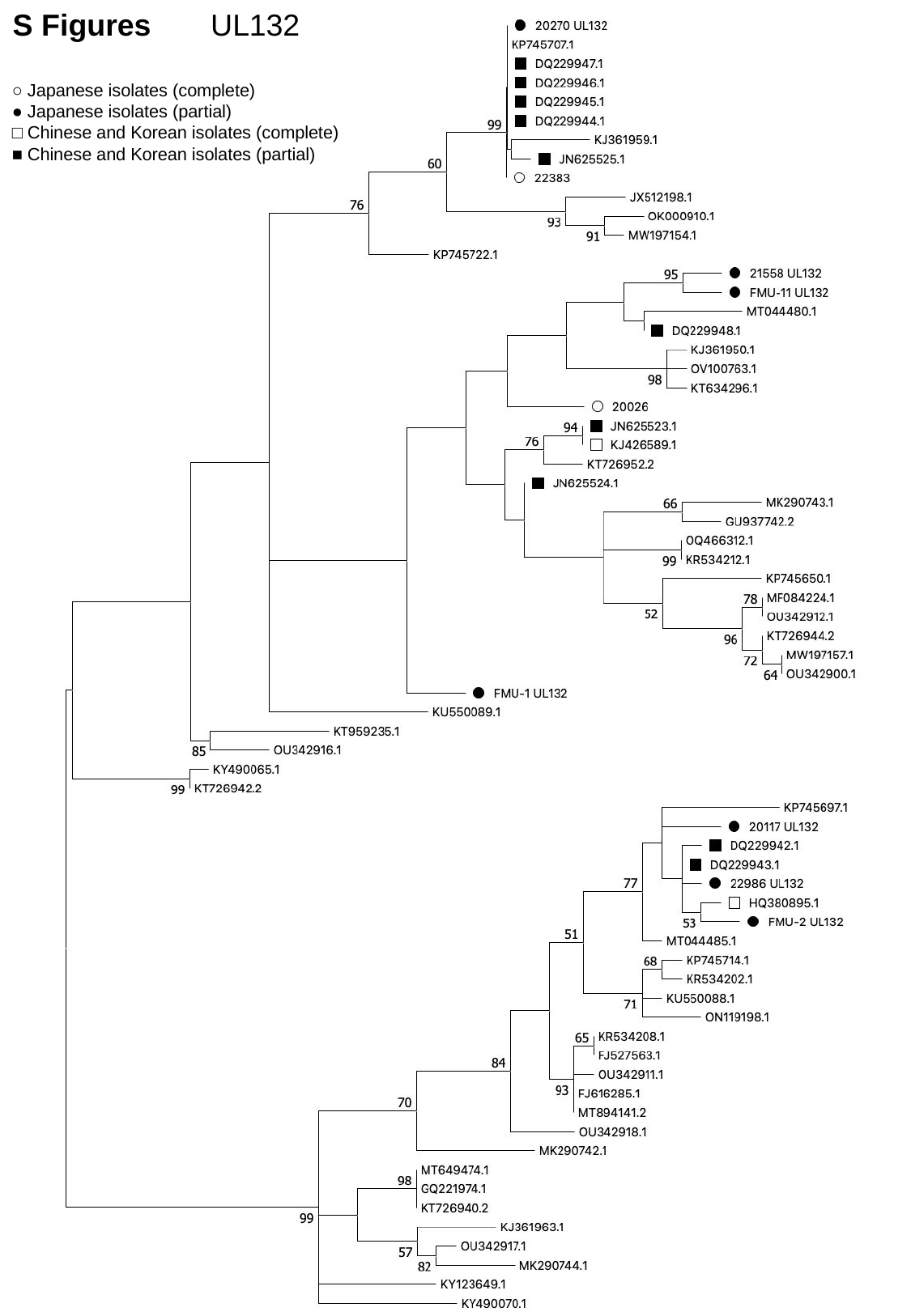

S Figures
UL132
○ Japanese isolates (complete)
● Japanese isolates (partial)
□ Chinese and Korean isolates (complete)
■ Chinese and Korean isolates (partial)

## Slide 5
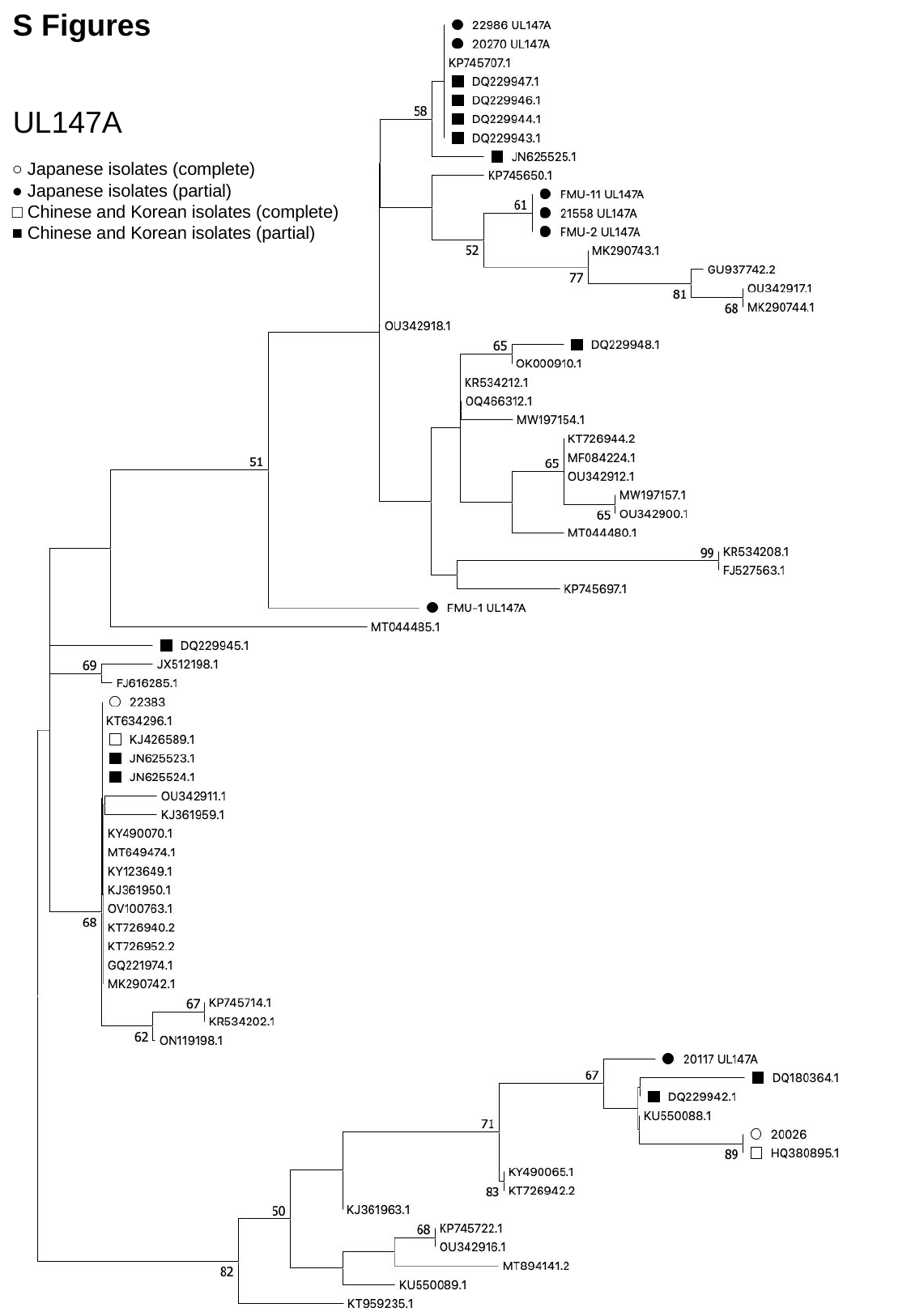

S Figures
UL147A
○ Japanese isolates (complete)
● Japanese isolates (partial)
□ Chinese and Korean isolates (complete)
■ Chinese and Korean isolates (partial)

## Slide 6
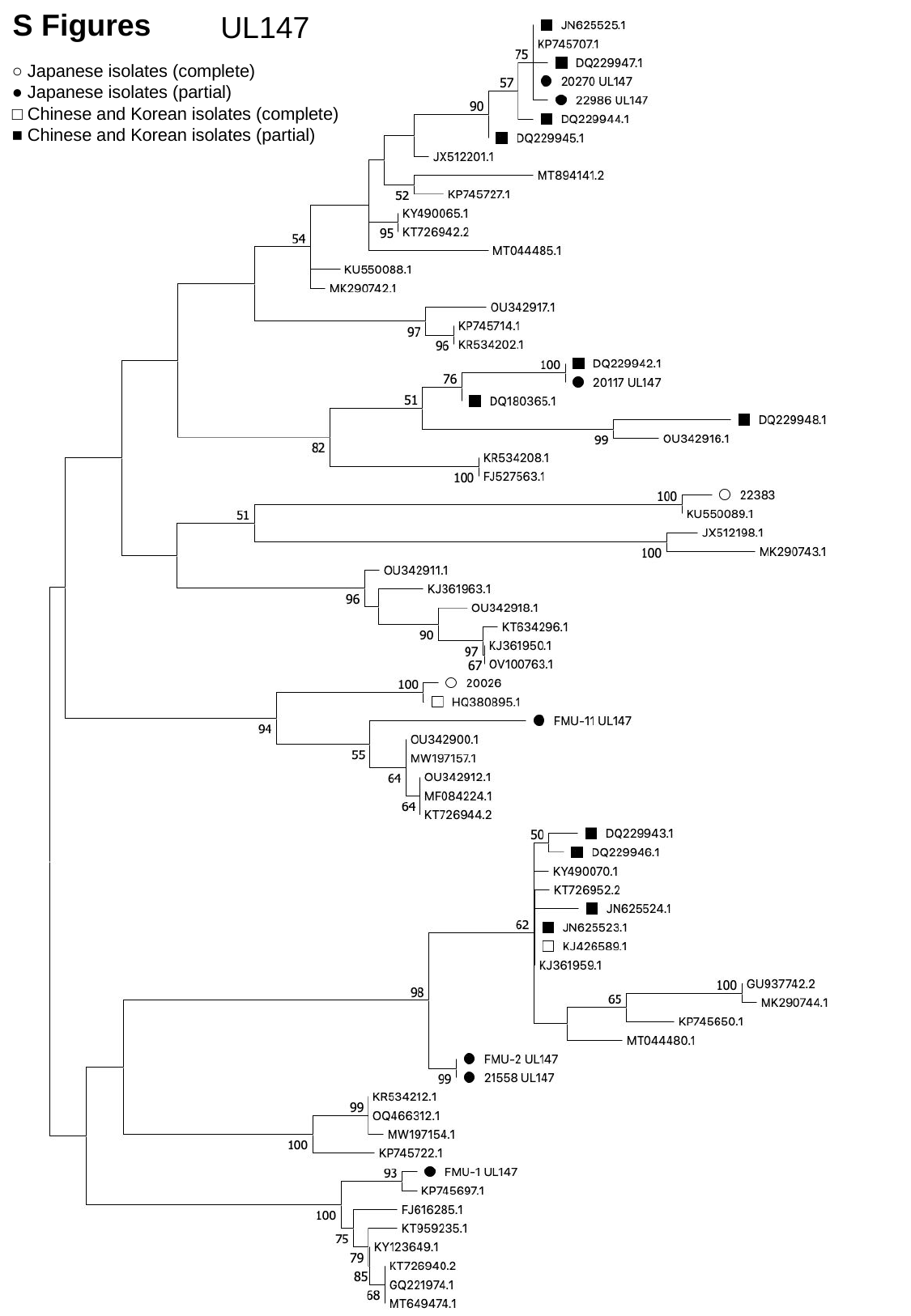

S Figures
UL147
○ Japanese isolates (complete)
● Japanese isolates (partial)
□ Chinese and Korean isolates (complete)
■ Chinese and Korean isolates (partial)

## Slide 7
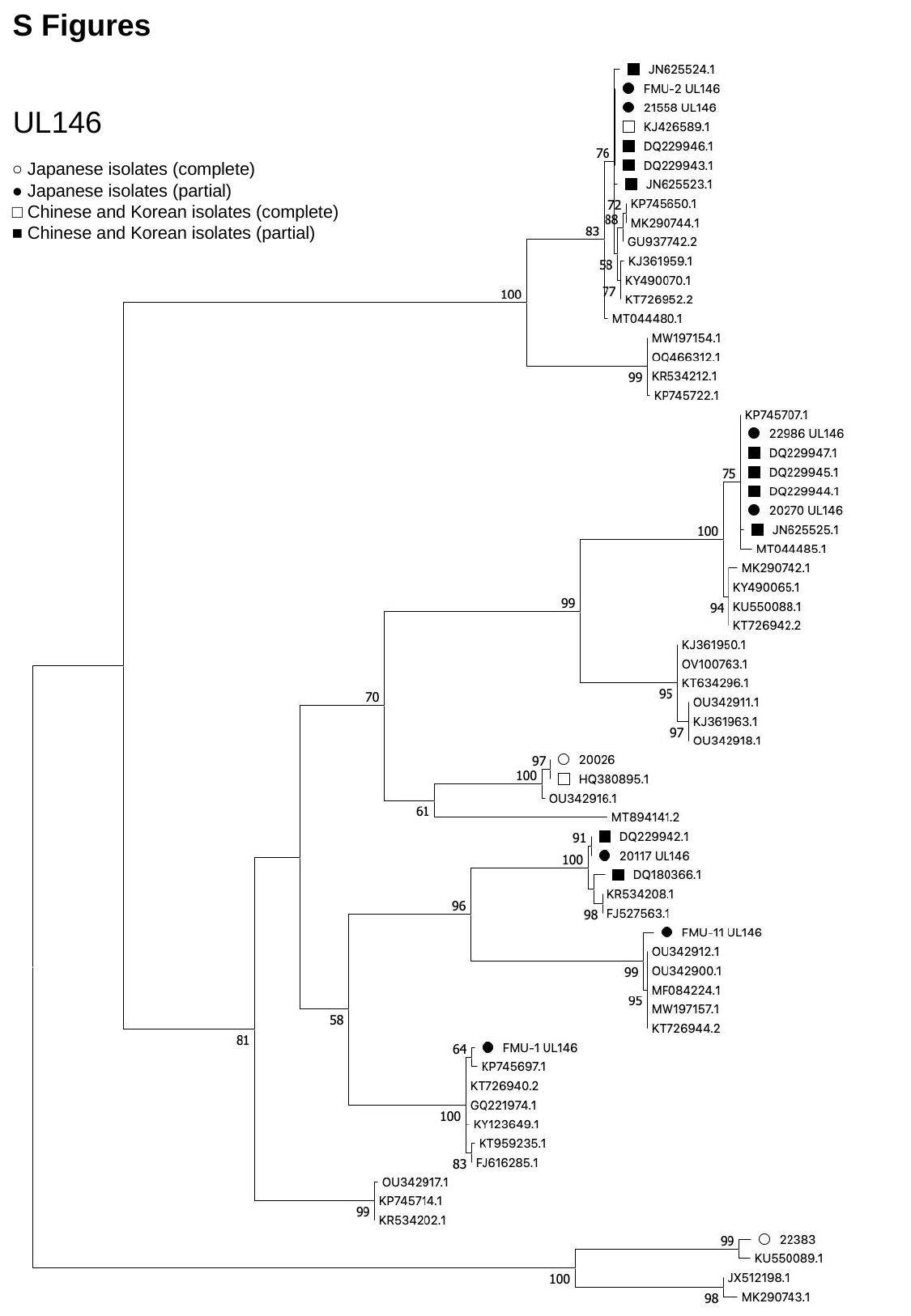

S Figures
UL146
○ Japanese isolates (complete)
● Japanese isolates (partial)
□ Chinese and Korean isolates (complete)
■ Chinese and Korean isolates (partial)

## Slide 8
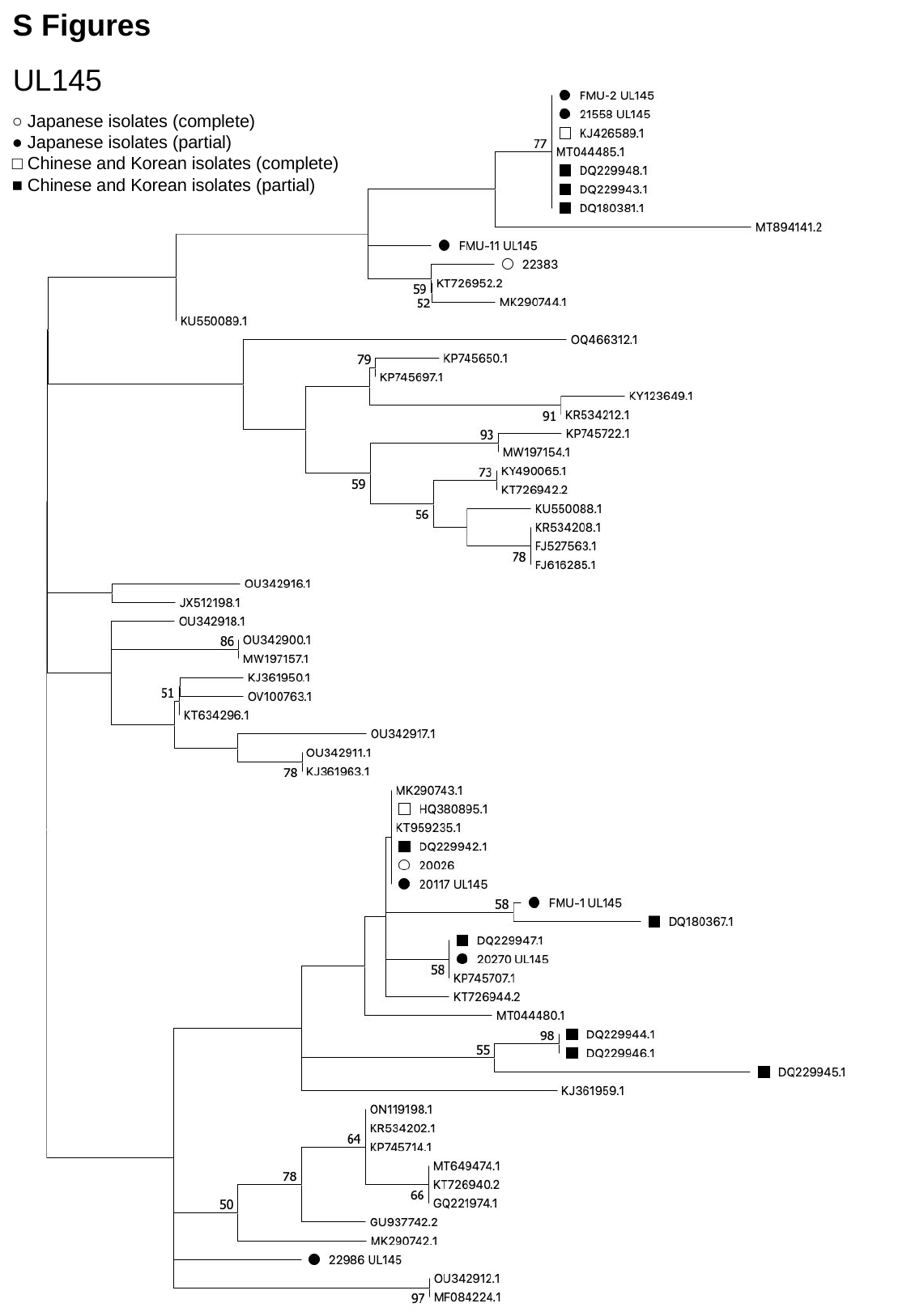

S Figures
UL145
○ Japanese isolates (complete)
● Japanese isolates (partial)
□ Chinese and Korean isolates (complete)
■ Chinese and Korean isolates (partial)

## Slide 9
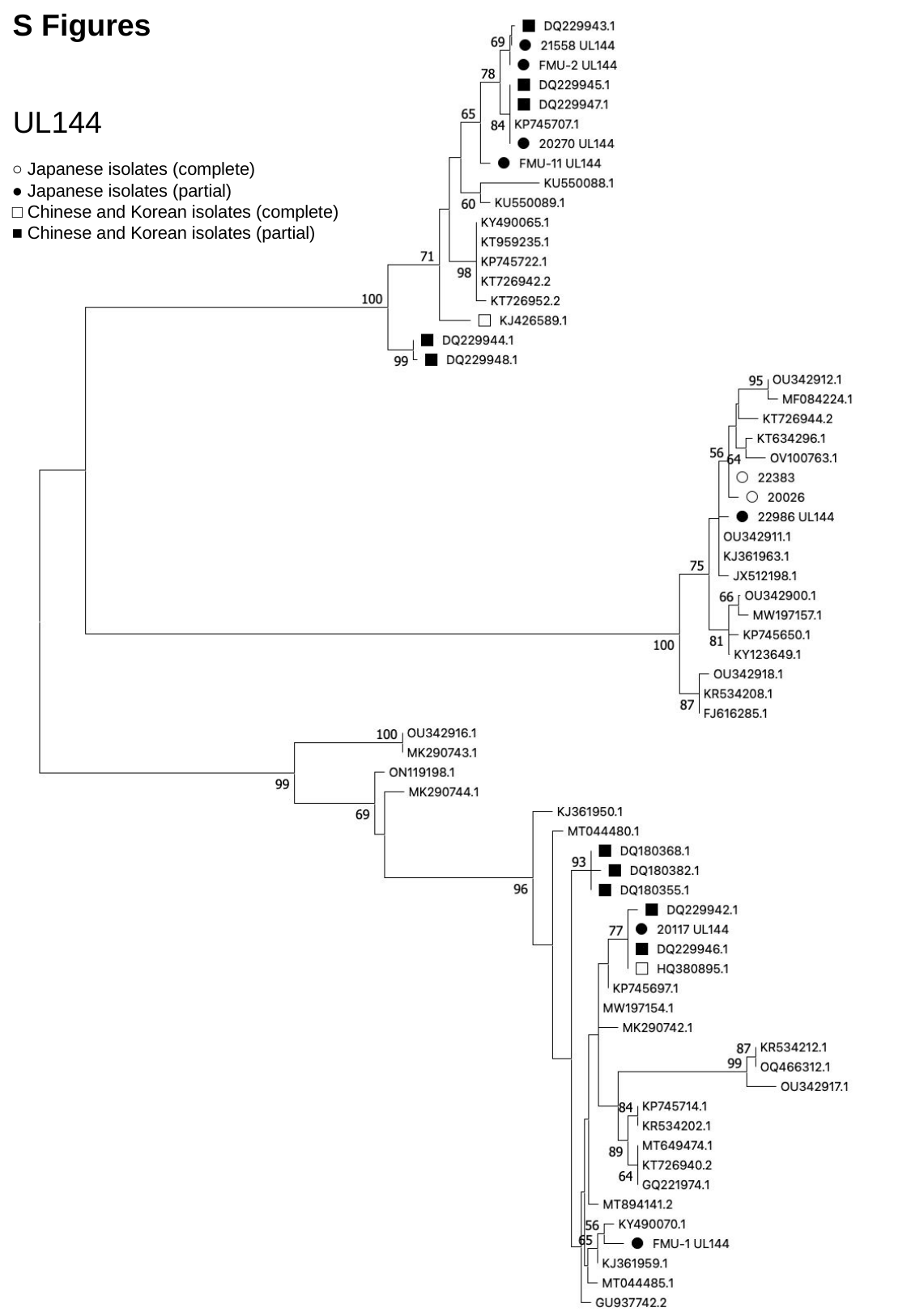

S Figures
UL144
○ Japanese isolates (complete)
● Japanese isolates (partial)
□ Chinese and Korean isolates (complete)
■ Chinese and Korean isolates (partial)

## Slide 10
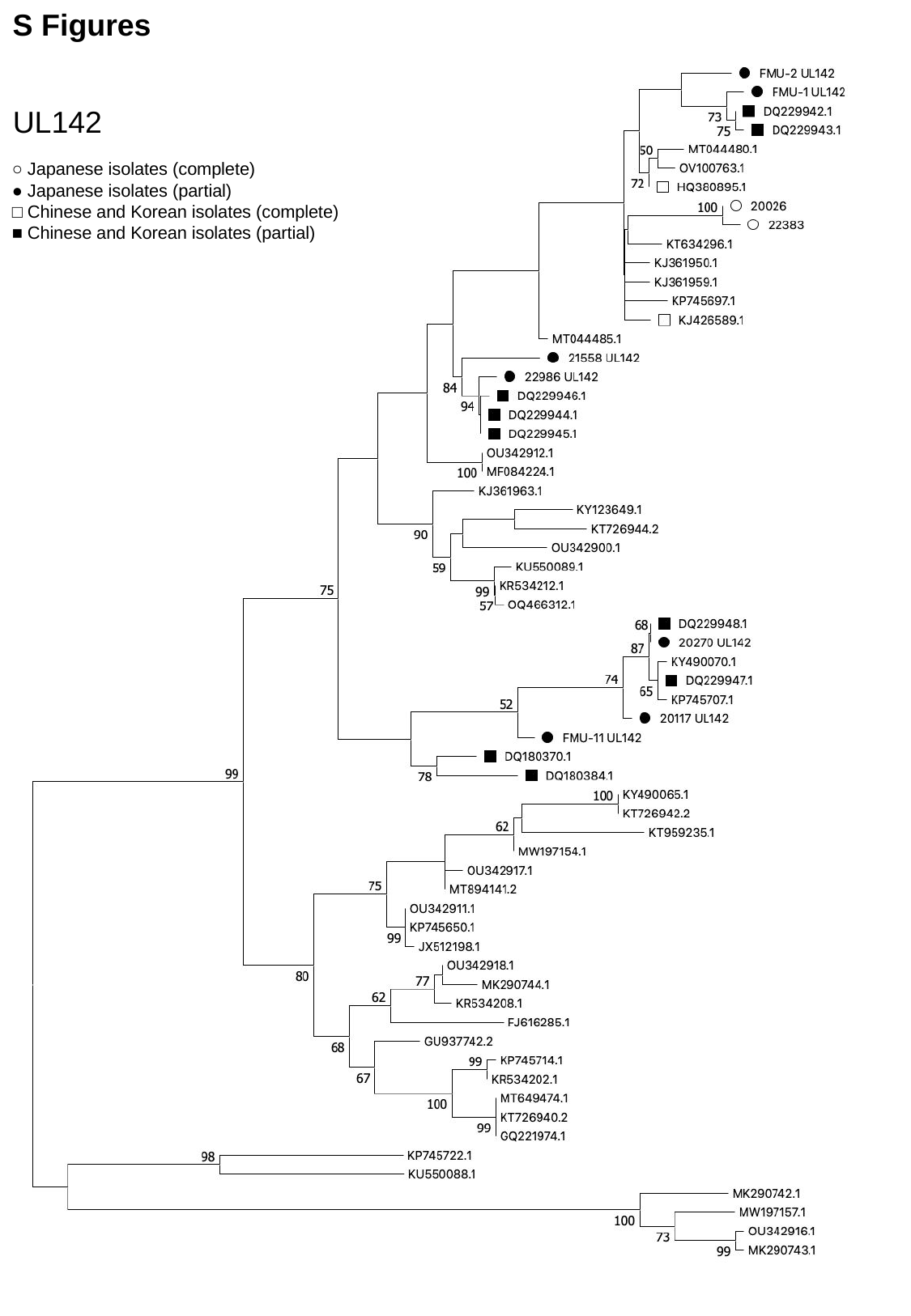

S Figures
UL142
○ Japanese isolates (complete)
● Japanese isolates (partial)
□ Chinese and Korean isolates (complete)
■ Chinese and Korean isolates (partial)

## Slide 11
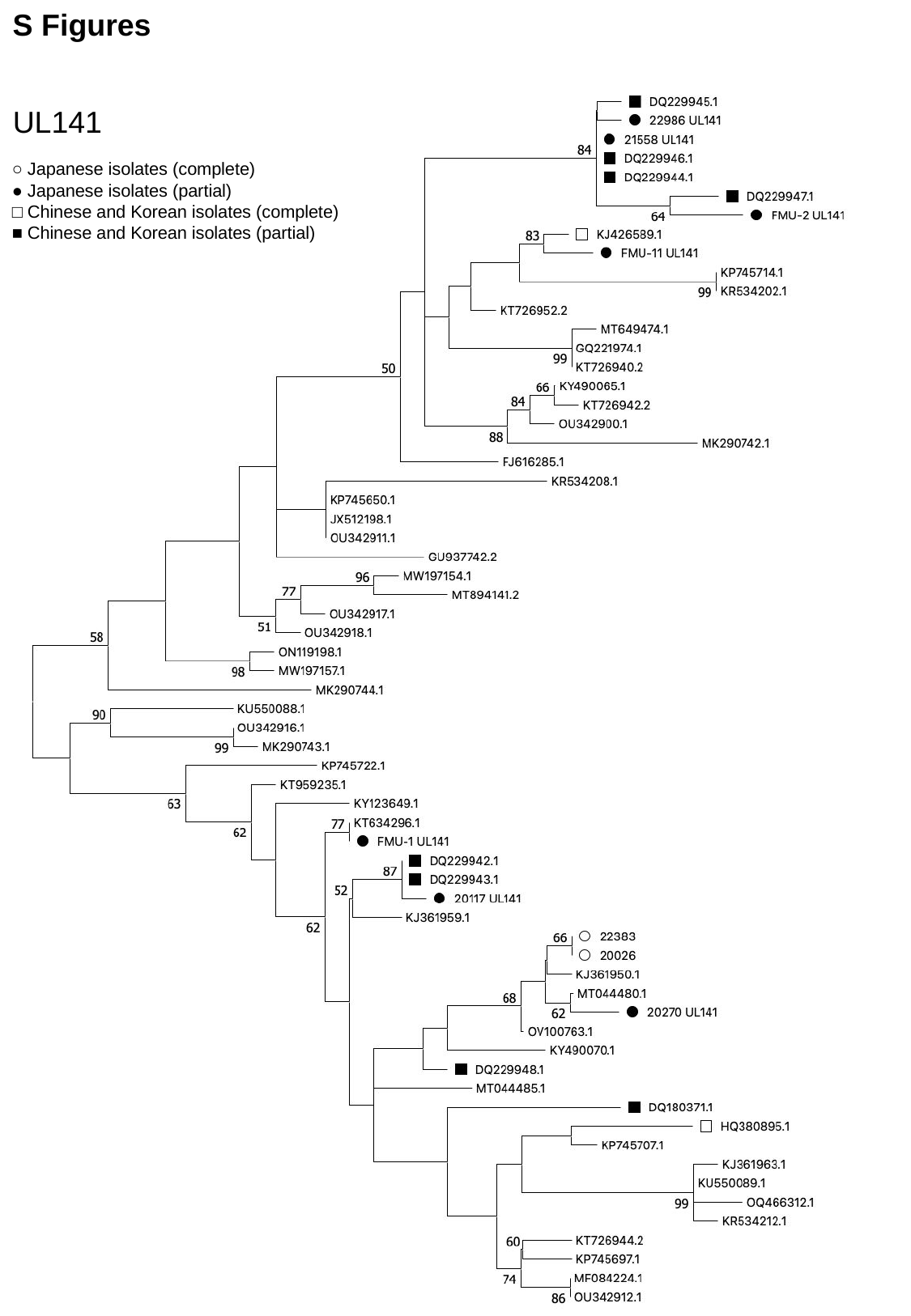

S Figures
UL141
○ Japanese isolates (complete)
● Japanese isolates (partial)
□ Chinese and Korean isolates (complete)
■ Chinese and Korean isolates (partial)

## Slide 12
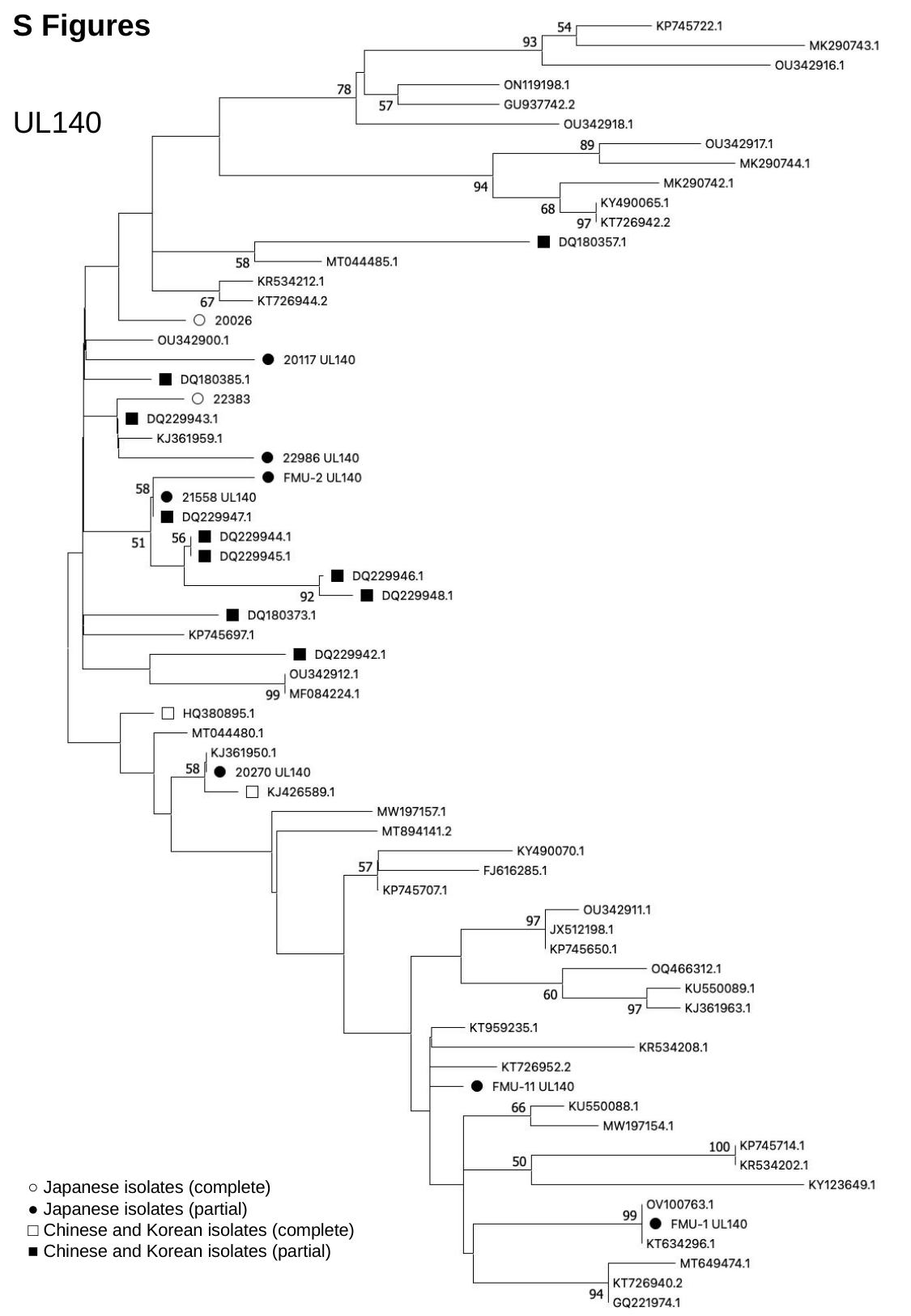

S Figures
UL140
○ Japanese isolates (complete)
● Japanese isolates (partial)
□ Chinese and Korean isolates (complete)
■ Chinese and Korean isolates (partial)

## Slide 13
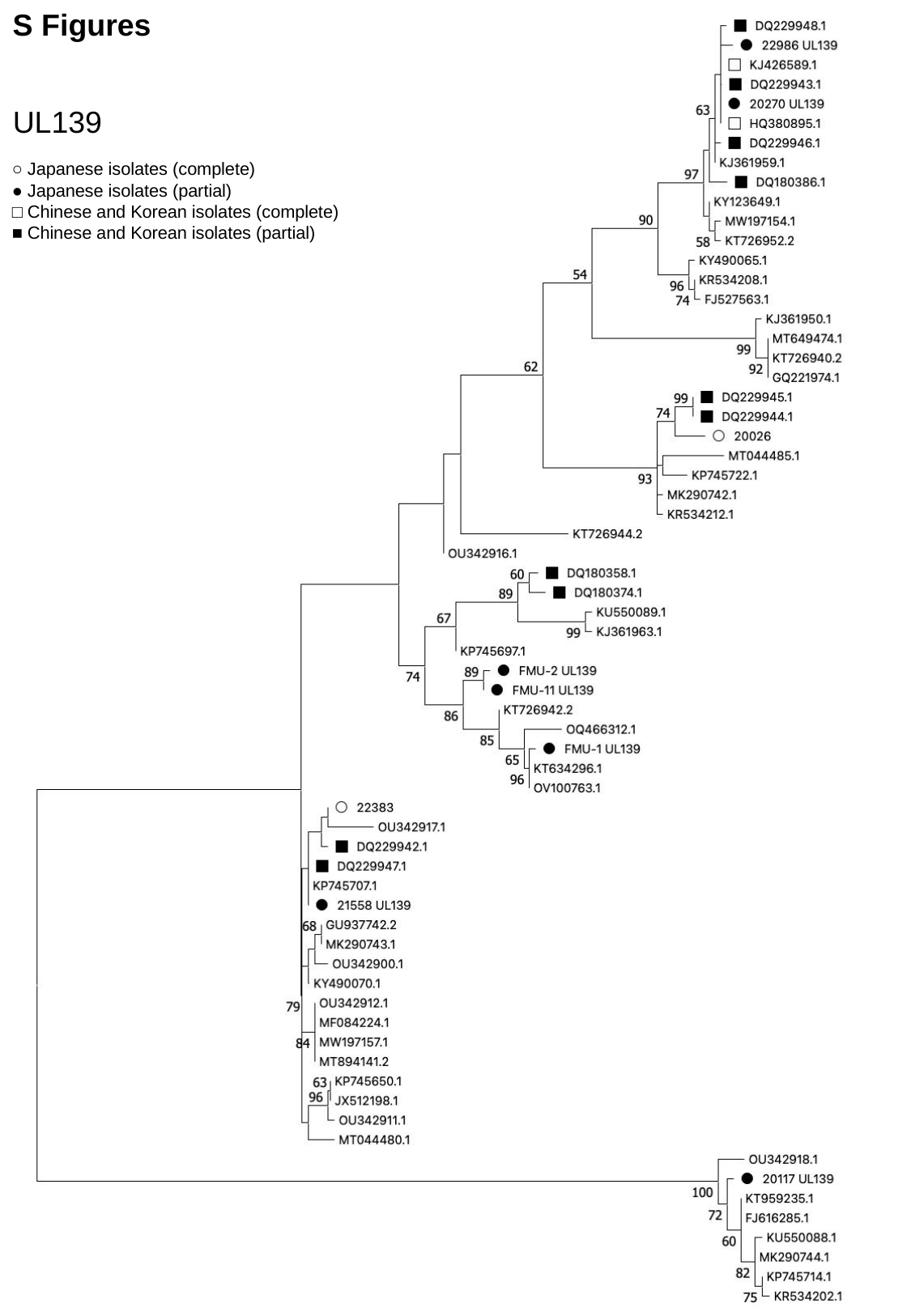

S Figures
UL139
○ Japanese isolates (complete)
● Japanese isolates (partial)
□ Chinese and Korean isolates (complete)
■ Chinese and Korean isolates (partial)

## Slide 14
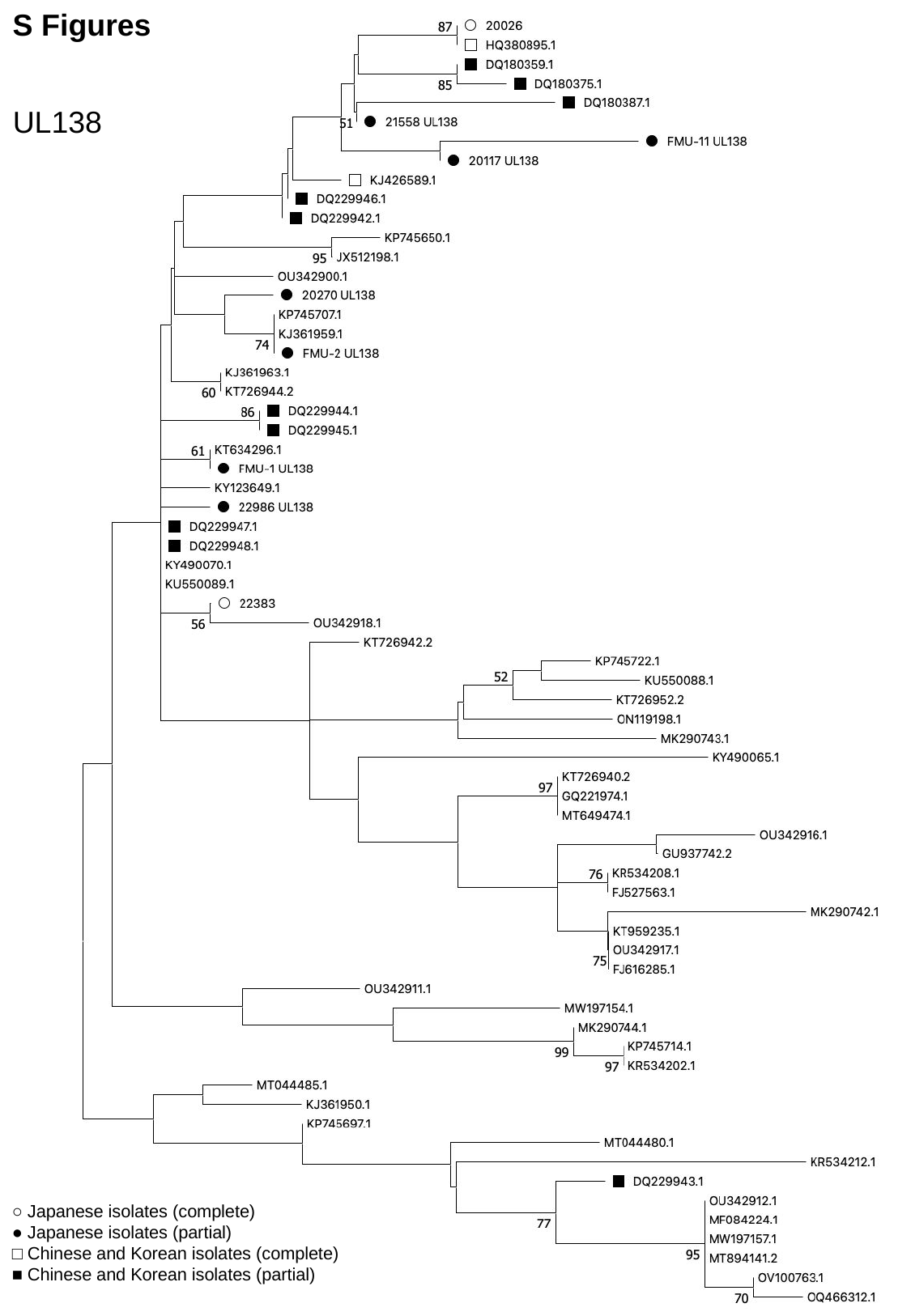

S Figures
UL138
○ Japanese isolates (complete)
● Japanese isolates (partial)
□ Chinese and Korean isolates (complete)
■ Chinese and Korean isolates (partial)

## Slide 15
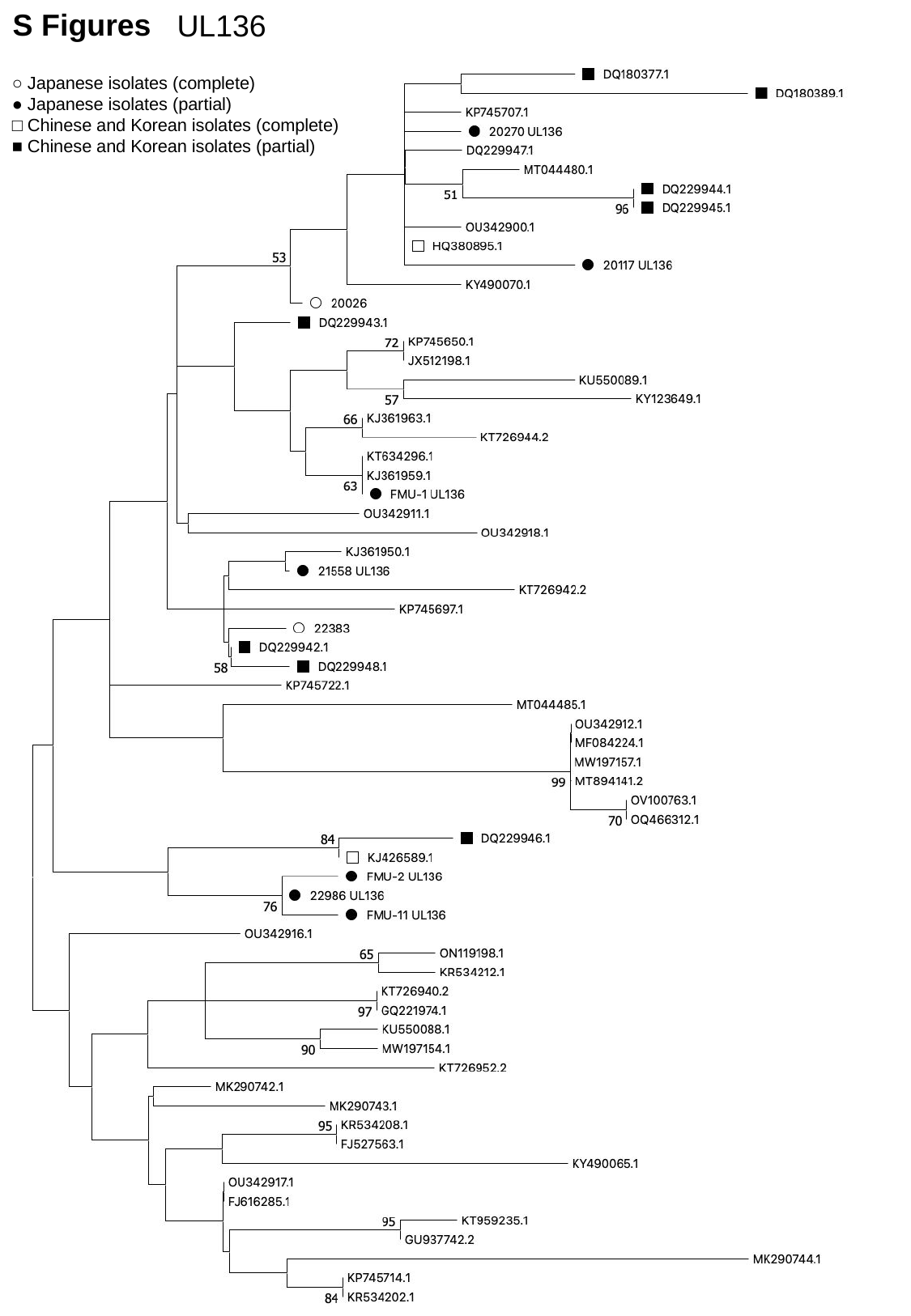

S Figures
UL136
○ Japanese isolates (complete)
● Japanese isolates (partial)
□ Chinese and Korean isolates (complete)
■ Chinese and Korean isolates (partial)

## Slide 16
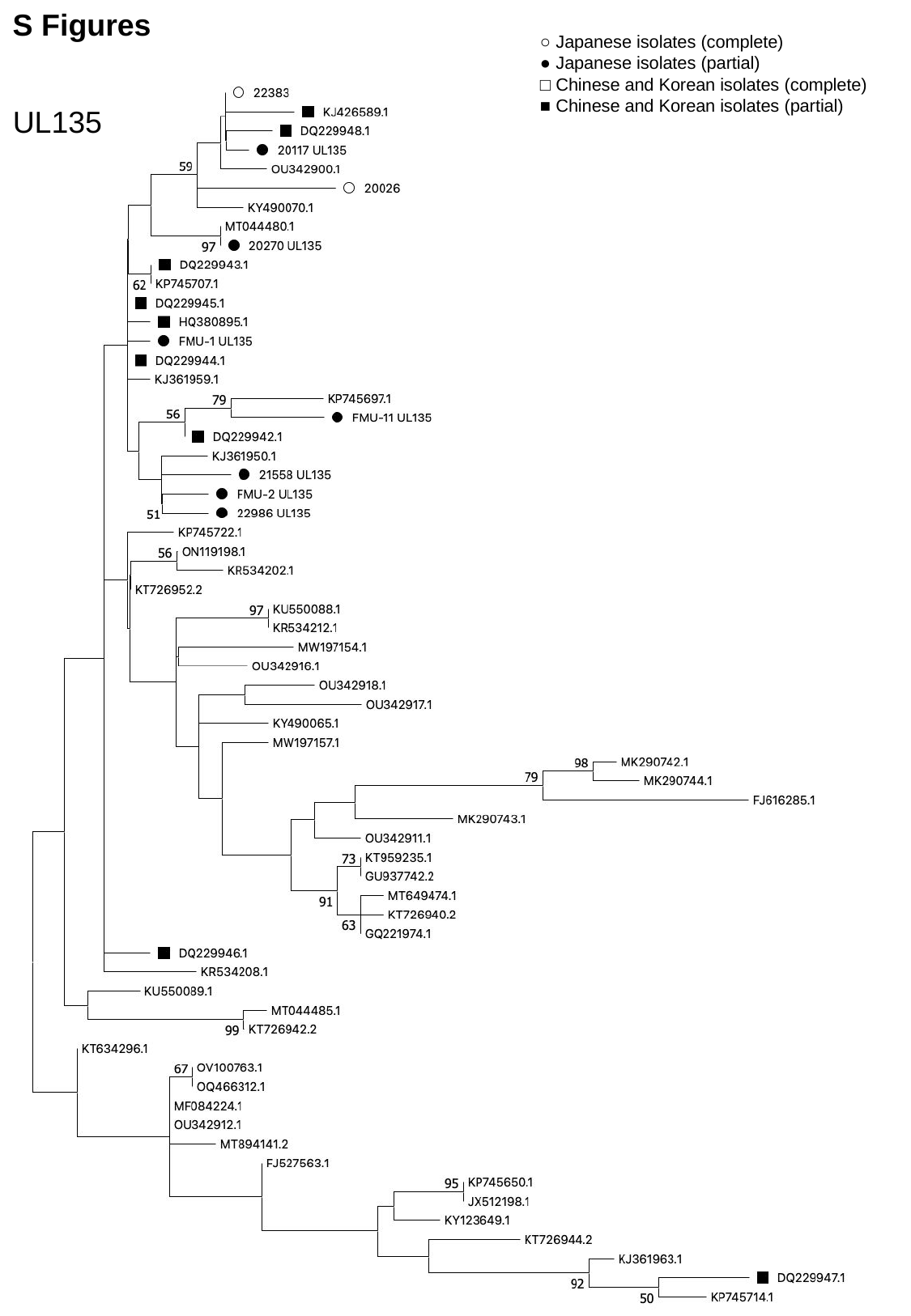

S Figures
○ Japanese isolates (complete)
● Japanese isolates (partial)
□ Chinese and Korean isolates (complete)
■ Chinese and Korean isolates (partial)
UL135

## Slide 17
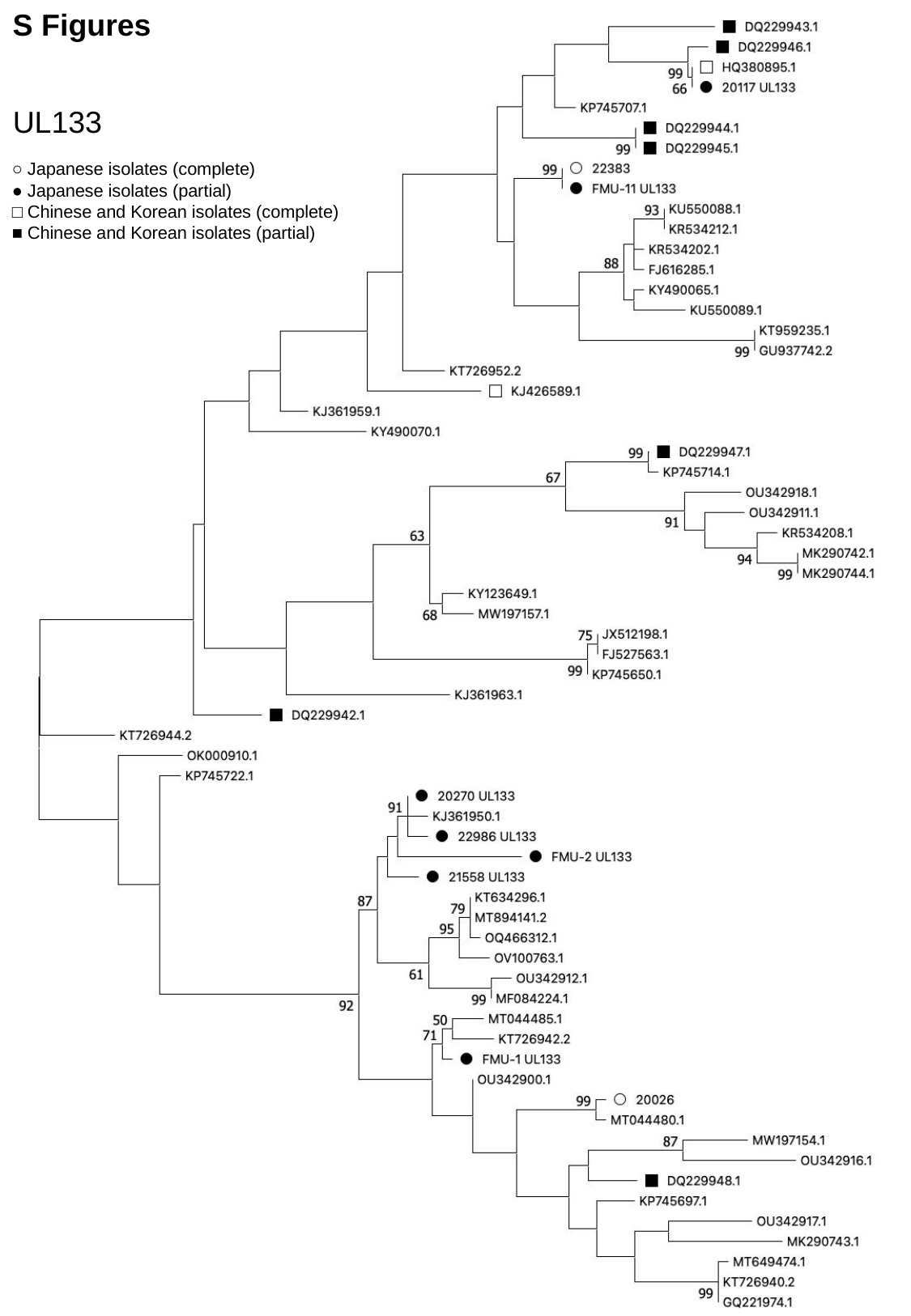

S Figures
UL133
○ Japanese isolates (complete)
● Japanese isolates (partial)
□ Chinese and Korean isolates (complete)
■ Chinese and Korean isolates (partial)

## Slide 18
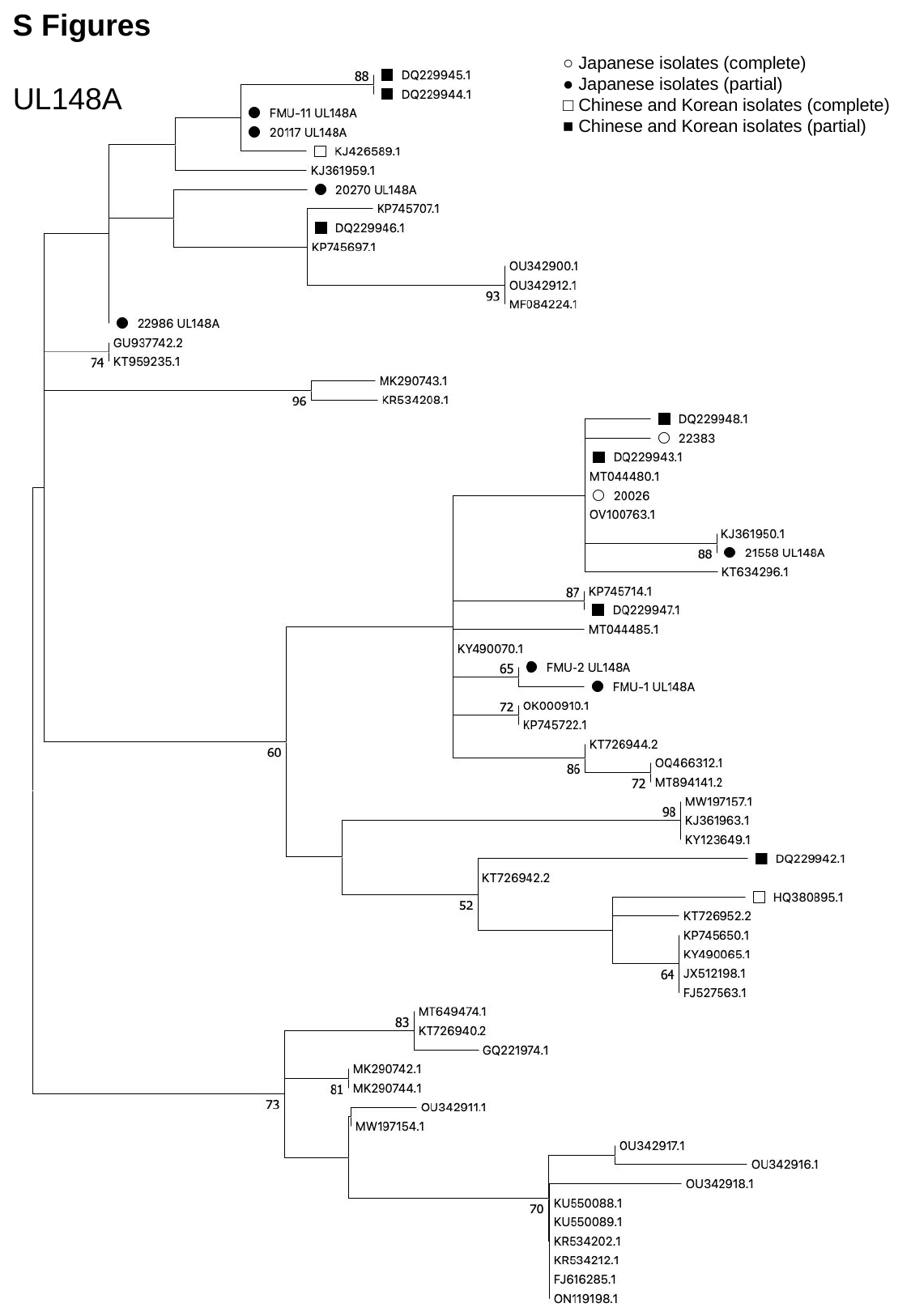

S Figures
○ Japanese isolates (complete)
● Japanese isolates (partial)
□ Chinese and Korean isolates (complete)
■ Chinese and Korean isolates (partial)
UL148A

## Slide 19
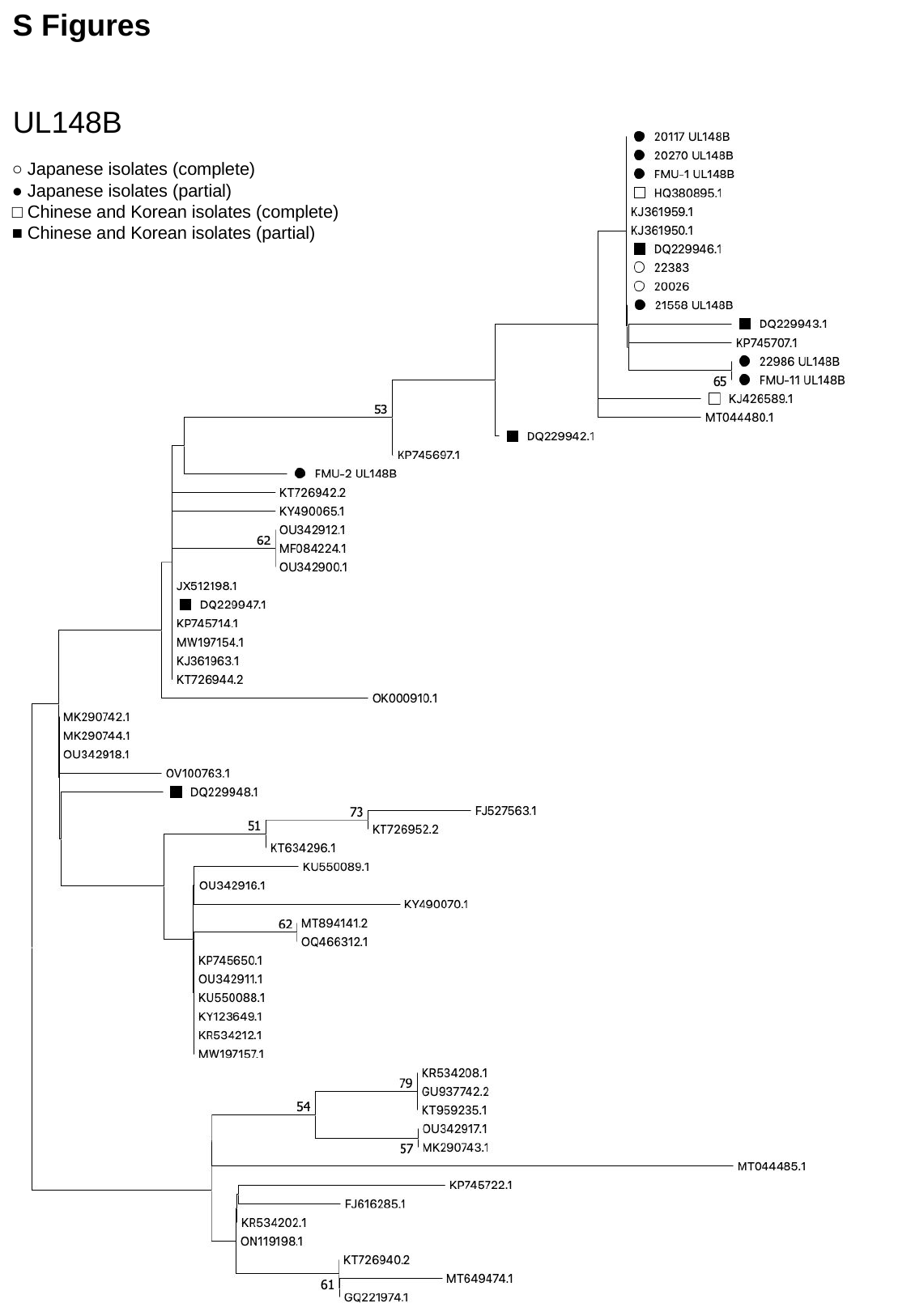

S Figures
UL148B
○ Japanese isolates (complete)
● Japanese isolates (partial)
□ Chinese and Korean isolates (complete)
■ Chinese and Korean isolates (partial)

## Slide 20
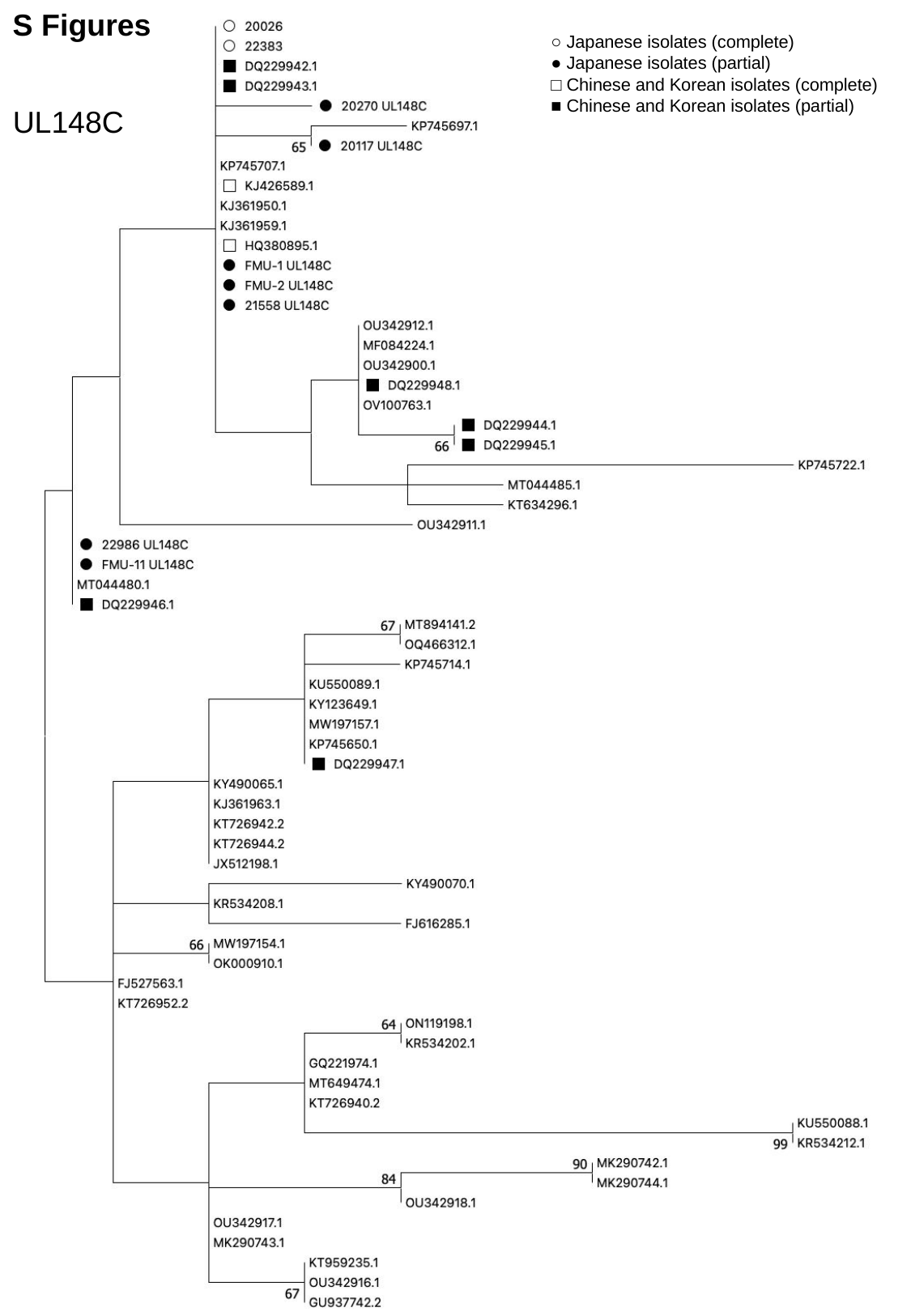

S Figures
○ Japanese isolates (complete)
● Japanese isolates (partial)
□ Chinese and Korean isolates (complete)
■ Chinese and Korean isolates (partial)
UL148C

## Slide 21
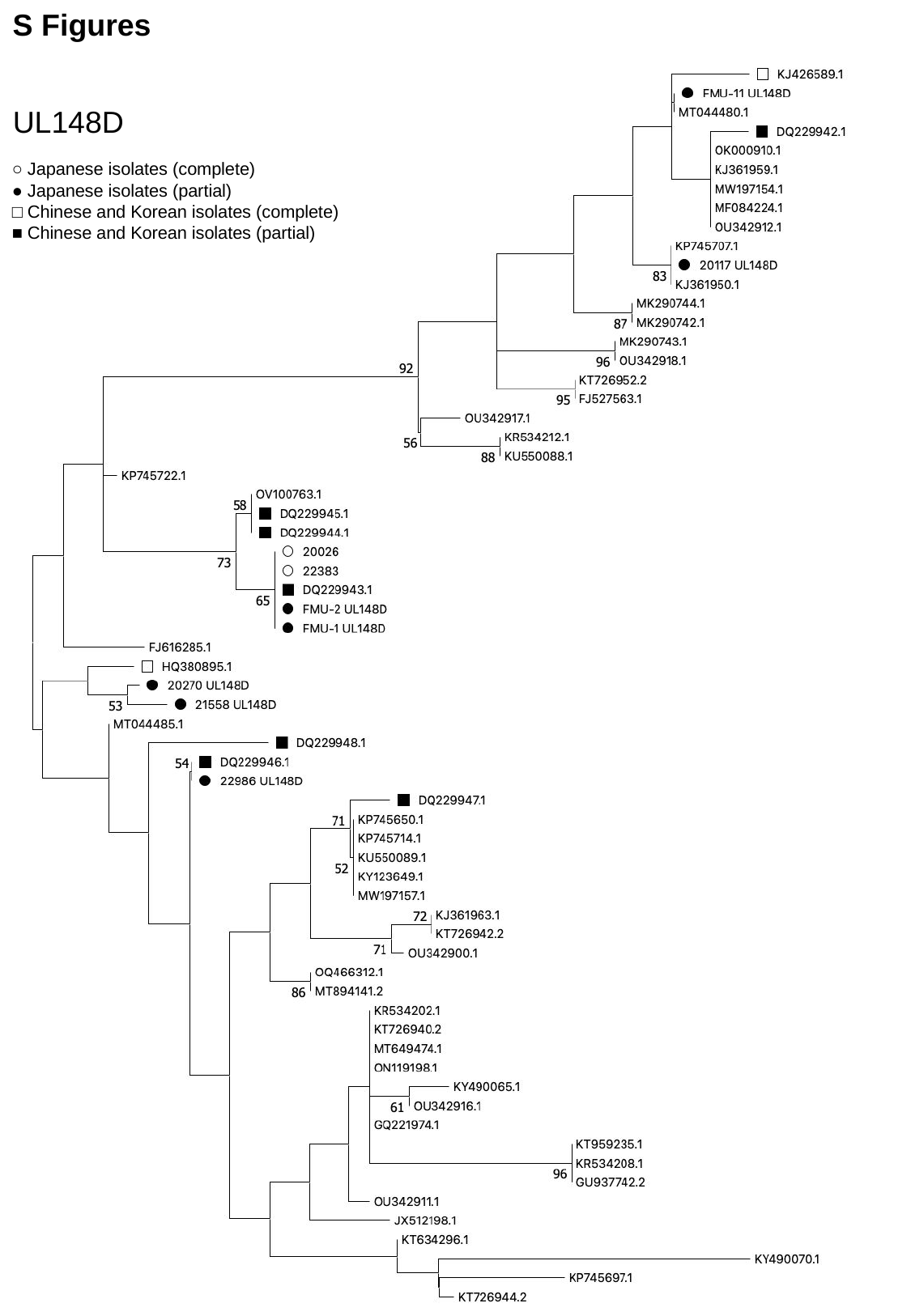

S Figures
UL148D
○ Japanese isolates (complete)
● Japanese isolates (partial)
□ Chinese and Korean isolates (complete)
■ Chinese and Korean isolates (partial)

## Slide 22
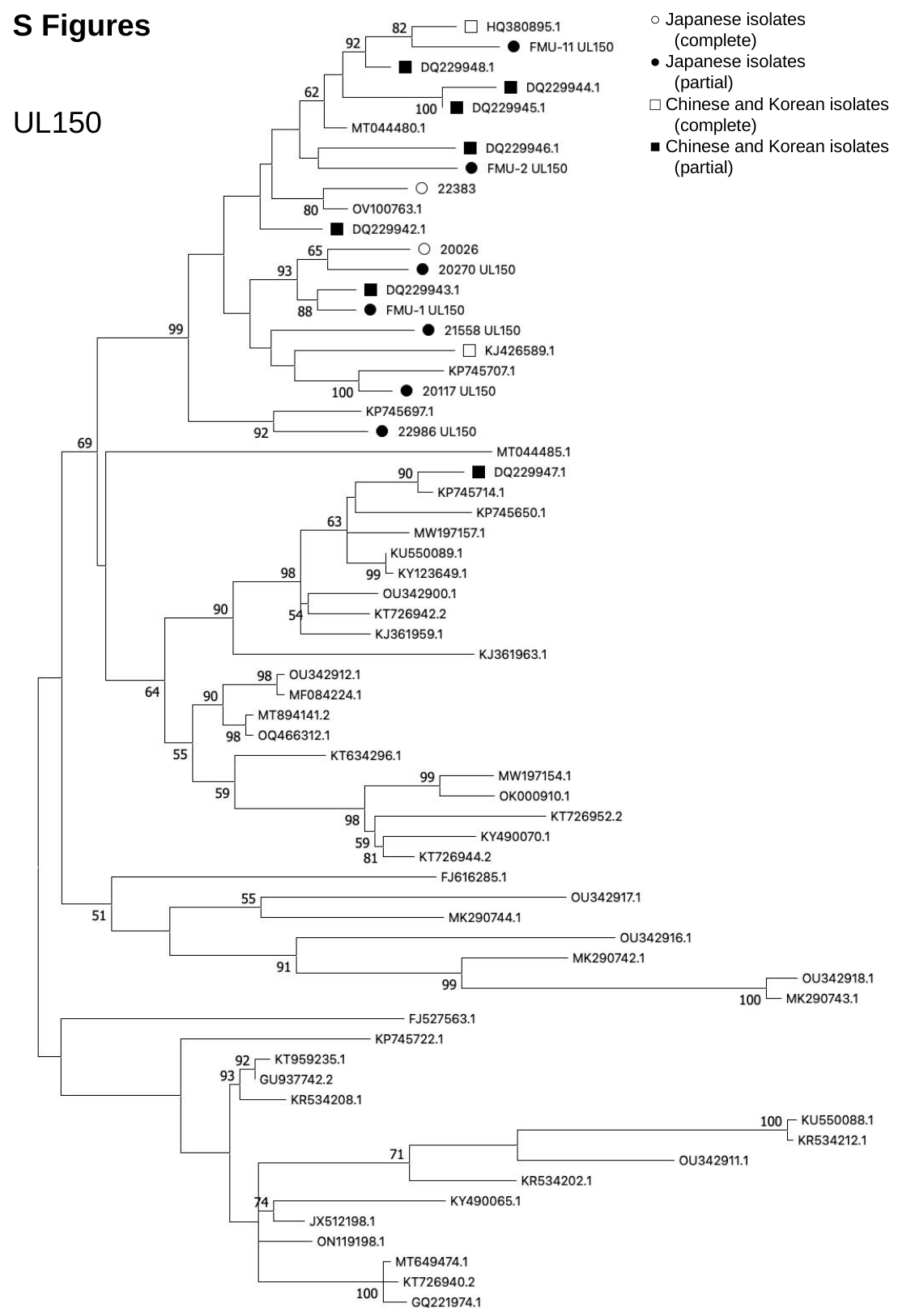

S Figures
○ Japanese isolates
 (complete)
● Japanese isolates
 (partial)
□ Chinese and Korean isolates
 (complete)
■ Chinese and Korean isolates
 (partial)
UL150

## Slide 23
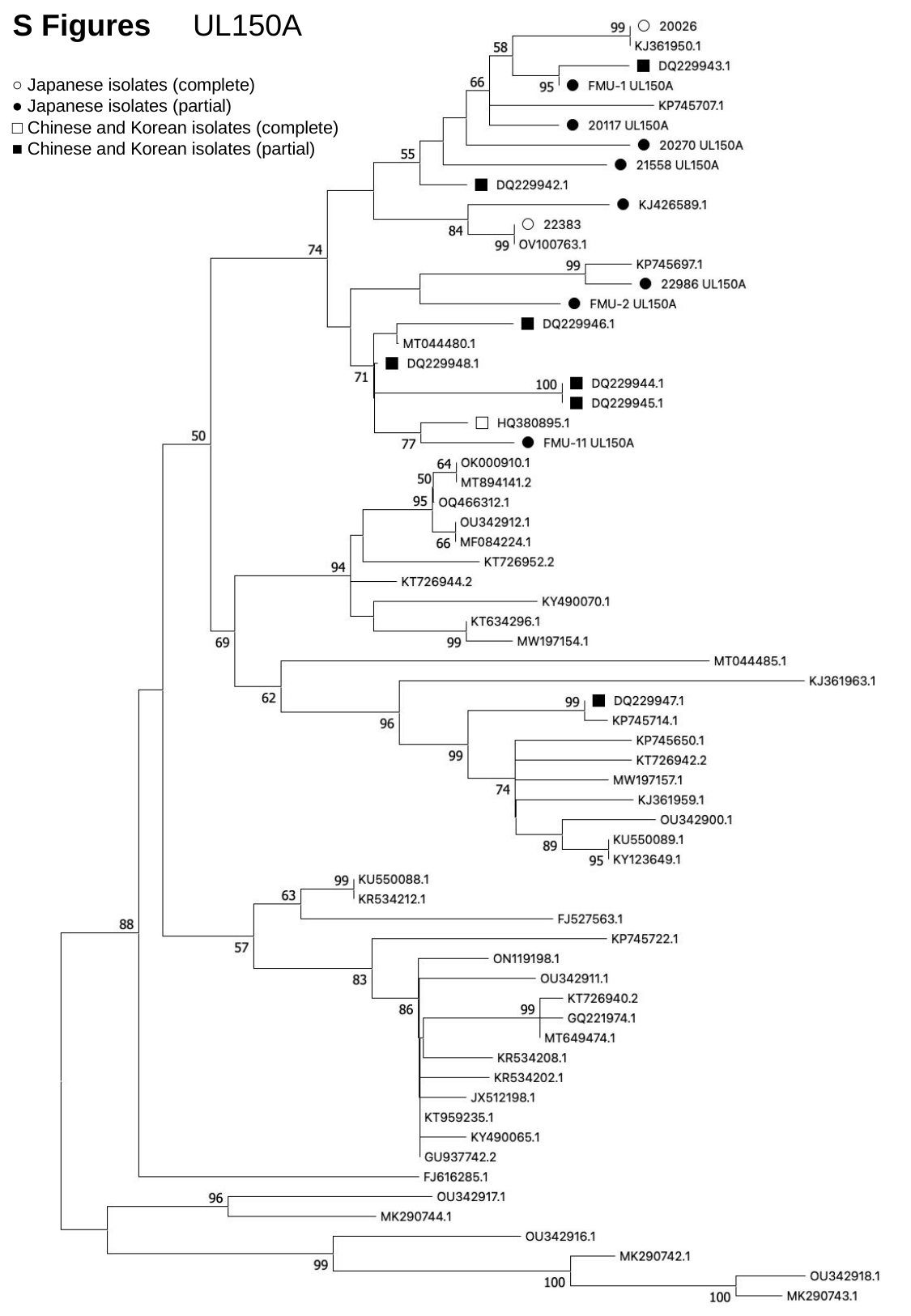

S Figures
UL150A
○ Japanese isolates (complete)
● Japanese isolates (partial)
□ Chinese and Korean isolates (complete)
■ Chinese and Korean isolates (partial)
