# Supplementary material for: Determination of whole genome sequence of human cytomegalovirus circulating in Japan and discovery of geographic genome structure in UL148 gene
Source: Virus Res. 2025 Feb 4;353:199540. doi: 10.1016/j.virusres.2025.199540 (PMC11846927; doi:10.1016/j.virusres.2025.199540)
Supplement: Supplementary file 2 [file mmc2.docx]

**Supplemental Figure Legends**

**Phylogenetic analysis of HCMV genes in UL/b' region.**

Phylogenetic trees of 23 genes in the UL/b' region were constructed using nucleotide sequences of HCMV by Maximum Likelihood phylogenetic analysis in the MEGA11 program. The percentage of trees in which the associated taxa clustered together was calculated by 1,000 replicates, and the frequencies of >50% were shown at the branches. The two sequences derived from whole genome sequences of HCMV determined in this study are depicted by white circle. The other seven sequences determined in this study are depicted by black circle. The deposited sequences in GenBank which, were isolated in China and Korea, were depicted by white (complete sequences) and black (partial sequences) squares, respectively.
